# Strategic hotel management for inclusive tourism: improving accessibility, service quality, and competitive advantage

**DOI:** 10.3389/fspor.2026.1806470

**Published:** 2026-07-08

**Authors:** Sarvinoz Atoevna Toyirova, Asliddin Junaydullayevich Abdulloyev, Hilola Abdullayeva, Nozima Giyazova, Mels Junaydullayev, Zebiniso Bakhtiyorovna Navruz-zoda, Sitora Islamovna Xasanova, Nigina Odinayeva

**Affiliations:** 1 Faculty of Economics and Tourism, Bukhara State University, Bukhara, Uzbekistan; 2 Tourism and Hotel Management Department, Faculty of Economics and Tourism, Bukhara State University, Bukhara, Uzbekistan

**Keywords:** accessibility, competitive advantage, hotel management, inclusive tourism, service quality, strategic management, universal design

## Abstract

Inclusive tourism represents a significant shift in hospitality management, one that requires the strategic integration of accessibility, service excellence, and competitive positioning. This conceptual study examines how hotel firms may cultivate enduring competitive advantages through inclusive practices that serve a broad range of visitor groups, including travelers with disabilities, senior travelers, families with young children, and culturally diverse guests. Drawing on strategic management theory, service quality frameworks, and disability studies, the paper develops an Inclusive Tourism Strategic Framework (ITSF) that connects accessibility management, service quality enhancement, and competitive strategy. The framework is constructed through a narrative review and theory-building synthesis of existing literature rather than through original empirical data. The analysis argues that inclusive tourism practices can generate several streams of value, including market expansion, brand strengthening, regulatory compliance, innovation, and sustained competitive advantage. The paper systematically examines accessibility management across physical, service, communication, and attitudinal dimensions; service quality enhancement through an adapted SERVQUAL model; and the mechanisms through which competitive advantage may be generated. It contends that inclusion is a strategic imperative rather than solely a social responsibility, and it offers conceptual guidance for hotel managers, policymakers, and tourism educators. The paper proposes that hotels which develop strong inclusive tourism capabilities are likely to achieve higher guest satisfaction, greater employee engagement, stronger stakeholder relationships, and improved financial performance, and it sets out a series of propositions to guide future empirical testing. The study contributes to hotel management literature by synthesizing previously separate theoretical perspectives into a unified strategic framework while offering practical guidance for industry professionals.

## 1 Introduction

The global tourism sector is at a turning point at which demographic change, technological development, and shifting societal values are converging to reshape how hospitality services are designed and delivered. Inclusive tourism, defined as tourism that enables people with accessibility needs across mobility, vision, hearing, and cognitive dimensions to participate autonomously, with equity and dignity, through universally designed tourism products, services, and environments (1), has emerged as both a moral obligation and a strategic opportunity (2, 3).

Current demographic trends underline the importance of inclusive tourism. The World Health Organization estimates that more than 1.3 billion people, or approximately 16% of the world's population, experience a significant disability (4). When family members and companions who travel with people requiring assistance are included, the inclusive tourism market is frequently estimated to encompass between 2.5 and 3 billion potential customers. In the European Union alone, accessible tourism has been estimated to generate in excess of €786 billion in annual economic activity (5). These figures are widely expected to grow as populations age and awareness increases, although the size and value of the market vary considerably across destinations and remain subject to methodological debate (6).

Beyond demographic considerations, inclusive tourism aligns with broader sustainability agendas. The United Nations Sustainable Development Goals, in particular Goal 10 (Reduced Inequalities) and Goal 11 (Sustainable Cities and Communities), call for tourism development that is both inclusive and durable. International instruments, including the UN Convention on the Rights of Persons with Disabilities and, more recently, ISO 21902:2021 on accessible tourism for all, establish legal and ethical standards that tourism operators are increasingly expected to meet (7).

Despite growing recognition, substantial gaps persist in hotel implementation. Studies repeatedly identify obstacles such as inadequate physical infrastructure, insufficient staff training, limited managerial knowledge, technological deficiencies, and persistent attitudinal barriers (8, 9). These issues are not solely a function of limited resources; they also reflect a more fundamental mismatch between conventional hospitality business models and the requirements of inclusive tourism.

The central problem addressed in this paper concerns the strategic integration of inclusive tourism principles into hotel management systems. Specifically, how can hotel firms systematically develop, implement, and sustain inclusive tourism strategies that simultaneously enhance accessibility, raise service quality, and create competitive advantage?

This research challenge manifests in several ways. Strategically, hotel managers often regard inclusive tourism as a cost center rather than a strategic investment (10), which leads to limited resource allocation and compliance-driven practices with modest reach. Implementation requires coordination across multiple functions, including front office, housekeeping, food and beverage, maintenance, security, and marketing, yet organizational silos and fragmented approaches impede effectiveness. From a knowledge standpoint, few theoretical frameworks combine accessibility management, service quality, and competitive strategy, leaving practitioners without integrated conceptual models. Finally, organizations frequently lack robust monitoring mechanisms and therefore cannot determine whether their inclusive tourism programs are succeeding.

Addressing these challenges carries both theoretical and practical significance. Recent scholarship has substantially advanced the accessible and inclusive tourism field, including syntheses of its conceptual foundations (11), perspective articles tracing its evolution (12), forward-looking agendas for the field (13), and bibliometric mappings of its development (14). Much of this work, however, treats accessibility primarily as a question of service provision, ethics, or destination policy. The present study extends this literature in three principal ways. First, it explicitly integrates competitive strategy, through the resource-based view and dynamic capabilities, with accessibility management, reframing inclusion as a potential source of sustainable competitive advantage rather than a compliance cost. Second, it extends the SERVQUAL model with two inclusive-tourism-specific dimensions, dignity preservation and proactive anticipation, rather than importing a generic service quality model unchanged. Third, it specifies a multi-level implementation architecture that addresses not only what accessibility requires but how hotels can develop, sequence, and sustain the relevant capabilities. Together, these elements distinguish the proposed Inclusive Tourism Strategic Framework (ITSF) from prior accessible tourism and service quality frameworks.

This conceptual study pursues four objectives. First, it synthesizes ideas from strategic management, service quality research, accessibility studies, and competitive advantage theory into a coherent conceptual model. Second, it develops the ITSF for hotel firms, identifying its principal capability domains, their interrelationships, and the mechanisms for implementation. Third, it examines how inclusive tourism practices may generate durable competitive advantage through differentiation, market expansion, brand enhancement, and innovation. Fourth, it derives practical recommendations addressing organizational capabilities, resource requirements, implementation sequencing, and performance measurement.

### 1.1 Methodological approach

This paper is a conceptual analysis and does not involve original empirical data collection. The ITSF was developed through a narrative review and theory-building synthesis, following an iterative process. First, the authors reviewed literature across four bodies of work: accessible and inclusive tourism, service quality, strategic management, and disability studies. Sources were identified through searches in Scopus, Web of Science, and Google Scholar, supplemented by backward and forward citation tracking of seminal works. Selection criteria for inclusion were the following: (i) peer-reviewed journal articles, scholarly monographs, or authoritative reports from internationally recognized organizations (for example, the World Health Organization, the European Commission, the International Organization for Standardization, and the United Nations); (ii) explicit engagement with at least one of the four target literatures, namely accessible or inclusive tourism, hospitality service quality, strategic management, or disability studies; (iii) theoretical or empirical contribution to the conceptual grounding of accessibility, inclusion, service quality, or competitive strategy in hospitality and tourism contexts; (iv) seminal foundational works [for example (15–17),] together with contemporary contributions, predominantly from 2000 onwards, that reflect current developments in the field; and (v) publication in English or availability of an authoritative English translation. Search terms combined accessibility- and inclusion-related keywords (for example, “inclusive tourism”, “accessible tourism”, “disability tourism”, “universal design”) with hospitality- and strategy-related keywords (for example, “hotel management”, “service quality”, “competitive advantage”, “dynamic capabilities”). Conference abstracts without accompanying full papers and non-peer-reviewed gray literature without institutional standing were excluded. Second, established theoretical lenses were selected on the basis of their explanatory relevance to the strategic integration of accessibility, with priority given to frameworks that are widely adopted and conceptually complementary. Third, concepts drawn from these lenses were compared, reconciled, and organized into a multi-level architecture, with attention to points of overlap and tension between disciplines. Fourth, the resulting synthesis was translated into testable propositions and into practitioner-oriented tools.

The framework, accessibility audit instrument, implementation roadmap, and performance indicators presented in the figures and tables are the authors' own conceptual elaborations. They are offered as analytical and planning tools to support reasoning and practice, and not as empirically validated measurement instruments. Their validation is identified as a priority for future research (see Sections 10 and 11).

## 2 Theoretical foundations

Contemporary inclusive tourism research draws on several theoretical frameworks. The universal design approach promotes the development of settings, products, and services that are accessible to all individuals to the greatest extent feasible, without the need for adaptation or specialized design (18–20). Universal design is guided by seven principles: equitable use, flexibility in use, simple and intuitive use, perceptible information, tolerance for error, low physical effort, and adequate size and space for approach and use. In hotel environments, universal design extends well beyond wheelchair ramps and accessible rooms to embody a comprehensive approach to the design of the guest experience.

A whole-of-life perspective acknowledges that access needs extend beyond permanent disability (21). Many people experience temporary or situational access requirements over the course of their lives, for example through pregnancy, injury, aging, childcare, or traveling with luggage. This perspective holds that inclusive design benefits all users rather than only specific disability-related segments. A market segmentation perspective highlights the commercial potential of inclusive tourism by recognizing several distinct groups, including people with disabilities, senior visitors, families with young children, and cultural groups with particular needs (1, 22). Understood as both an analytical concept and an aspirational goal, inclusive tourism development provides a lens for evaluating existing practice and guiding new development (3, 11). A rights-based perspective, grounded in human rights instruments and notably the UN Convention on the Rights of Persons with Disabilities, frames accessibility as a legal entitlement rather than a discretionary option (23). These complementary perspectives have evolved over time and continue to shape policy debates about how inclusion should be defined and operationalized in tourism practice (24, 25).

Service quality is a fundamental concept in hospitality management, with substantial research demonstrating the relationships among service quality, customer satisfaction, loyalty, and financial performance (26–28). The SERVQUAL framework (15, 29) identifies five dimensions of service quality: tangibles (physical facilities, equipment, and personnel appearance); reliability (the capacity to deliver promised services consistently and accurately); responsiveness (the readiness to assist customers and provide prompt service); assurance (the knowledge and courtesy of employees and their ability to inspire trust and confidence); and empathy (caring, individualized attention). Subsequent work has refined and extended these dimensions through hierarchical and multi-level reconceptualizations of perceived service quality (30), while service-dominant logic has reframed quality as value co-created between providers and customers (31). In the context of inclusive tourism, the SERVQUAL dimensions require adaptation to address accessibility-specific needs.

The service quality gaps model (15) identifies five potential discrepancies between customer expectations and perceptions: the knowledge gap, the standards gap, the delivery gap, the communications gap, and the customer gap. In inclusive tourism, further deficiencies arise in relation to accessibility awareness, the implementation of universal design, and staff competence. The critical incident approach (32) analyzes specific service interactions that strongly affect customer satisfaction, identifying decisive instances of service excellence or failure. For visitors with accessibility requirements, such incidents often occur when inclusive design enables independent functioning or when barriers cause frustration and exclusion. Research on service recovery indicates that an effective organizational response to service failures may raise customer satisfaction above the level attained when no problem occurs, a phenomenon known as the service recovery paradox (33), although empirical support for this effect is mixed and likely context-dependent.

Strategic management theory offers frameworks for understanding how firms develop and sustain competitive advantage in changing conditions. A differentiation strategy (34) treats distinctive value propositions as a primary competitive tactic, enabling firms to offer value that commands premium rates. Inclusive tourism practices can support differentiation through improved accessibility, elevated service quality, and demonstrable social responsibility. Stakeholder theory (35) holds that organizational performance depends on effectively managing relationships with multiple stakeholder groups, including customers, employees, suppliers, communities, regulators, and investors, and inclusive tourism can align these interests by creating value for several groups at once.

Building on earlier work that conceptualized the firm as a bundle of resources (36), the resource-based view (16) explains competitive advantage in terms of resources and capabilities that are valuable, rare, inimitable, and non-substitutable, summarized by the VRIN criteria. The dynamic capabilities perspective (17, 37, 38) extends this logic to changing environments, emphasizing the organizational capacity to sense opportunities, seize them, and reconfigure resources accordingly. Both perspectives are central to the present analysis, since they explain how inclusive tourism capabilities may become sources of durable rather than transient advantage. Table 1 summarizes the four theoretical perspectives that inform the framework developed in the subsequent sections.

**Table 1:** Theoretical perspectives informing inclusive tourism strategy.

Framework	Core concepts	Application	Key implications
Universal Design	Seven principles; design for all	Physical accessibility, service design	Holistic approach benefiting all guests
Social Model	Disability socially constructed	Removing environmental barriers	Focus on organizational responsibility
SERVQUAL	Five service quality dimensions	Adapted for accessibility requirements	Enhanced service quality measurement
Resource-Based View/Dynamic Capabilities	VRIN resources; sensing, seizing, reconfiguring	Inclusive capabilities as strategic resources	Sustainable competitive advantage

Authors’ own elaboration based on Parasuraman et al. (15), Barney (16), Teece et al. (17), and Center for Universal Design (18).

## 3 The inclusive tourism strategic framework (ITSF)

**Figure 1:**
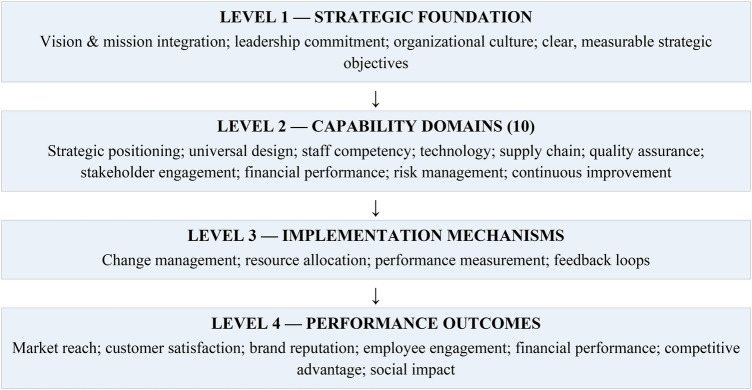
ITSF four-level architecture. Authors’ own elaboration based on strategic management and inclusive tourism literature.

Building on the theoretical foundations above, this section presents the Inclusive Tourism Strategic Framework (ITSF), a conceptual model designed to help hotel firms develop, implement, and sustain inclusive tourism practices that improve accessibility, enhance service quality, and create competitive advantage. As Figure 1 illustrates, the ITSF is organized into four levels: strategic foundation, capability domains, implementation mechanisms, and performance outcomes. This structure recognizes that inclusive tourism strategy operates within a dynamic environment shaped by evolving legislation, demographic change, technological development, shifting social values, and competitive pressure.

The ITSF builds on, and is intended to extend, existing accessible tourism frameworks. Earlier models have made important contributions in conceptualizing accessibility, mapping barriers, and positioning inclusion within destination development (2, 9, 11). The ITSF differs from these models in three respects. First, it places competitive strategy at the center of the analysis, treating inclusive capabilities explicitly as candidate VRIN resources rather than as service attributes or compliance obligations. Second, it integrates an extended service quality model within the same architecture, linking accessibility provision to perceived quality through the manner of service delivery. Third, it is an implementation-oriented framework, in that each level specifies not only desirable end states but the capabilities and mechanisms through which hotels can move toward them. The framework therefore synthesizes existing knowledge and seeks to advance it by connecting accessibility, service quality, and competitive advantage within a single, operationally specified model.

The strategic foundation establishes the organization's commitment to high-quality inclusive tourism. Integrating accessibility and inclusion into the organization's vision and mission signals to internal and external stakeholders that these values are core commitments rather than peripheral projects. Leadership commitment is decisive, since visible and sustained senior support shapes resource allocation, organizational priorities, and culture. Organizational culture, understood as the shared values, attitudes, and practices that guide behavior, strongly influences how effectively inclusive practices take hold. Strategic objectives translate the vision into specific, measurable goals, for example a target guest satisfaction rating among guests with accessibility needs to be achieved within a defined period.

The ITSF identifies ten capability domains that organizations draw on to deliver inclusive tourism excellence. Strategic positioning includes market analysis, competitive assessment, value proposition formulation, and stakeholder mapping. Universal design implementation covers buildings, service operations, communication systems, and the surrounding environment. Staff competency development includes training, awareness-raising, technical skill development, and attitudinal change. Technology integration addresses accessible digital content, assistive technology, booking systems, and information platforms. Supply chain management covers supplier selection, partner coordination, third-party services, and quality assurance. Quality assurance systems include performance monitoring, customer feedback, evaluation processes, and continuous improvement. Stakeholder engagement involves customers, employees, community partners, and advocacy organizations. Financial performance management covers revenue tracking, cost management, return-on-investment measurement, and value creation. Risk management includes compliance monitoring, liability management, reputation protection, and crisis response. Continuous improvement covers innovation methods, learning processes, the adoption of best practices, and knowledge management. Organizations need not excel in all domains simultaneously; the ITSF supports incremental development aligned with an organization's environment, resources, and goals, consistent with capability lifecycle perspectives in strategic management (38) and with destination-level accessibility research that emphasizes information, infrastructure, and competence as interdependent capability domains (39). Table 2 summarizes selected capability domains, their components, success factors, and primary outcomes.

**Table 2:** ITSF capability domain specifications (selected domains).

Domain	Key components	Success factors	Primary outcomes
Strategic Positioning	Market analysis, competitive assessment, value proposition	Deep market understanding; clear differentiation	Enhanced market position
Universal Design	Physical infrastructure, service processes, communication	Comprehensive accessibility; proactive design	Barrier-free experiences
Staff Competency	Training, awareness, skills, attitudes	Systematic training; behavioral change	Confident, capable staff
Technology Integration	Digital platforms, assistive technology, booking systems	Accessible technology; seamless integration	Enhanced convenience
Quality Assurance	Monitoring, feedback, assessment, improvement	Systematic measurement; guest input	Consistent quality

Authors’ own elaboration. The table is a conceptual planning tool; the full set of ten domains is described in the text.

### 3.1 Relationships among framework components

The four levels of the ITSF are connected by a directional logic. The strategic foundation (Level 1) is treated as foundational: leadership commitment, cultural values, and strategic objectives shape the resources allocated to, and the priority given to, capability development. The capability domains (Level 2) are the proximate drivers of inclusive tourism performance, while the implementation mechanisms (Level 3), namely change management, resource allocation, performance measurement, and feedback loops, mediate the translation of capabilities into outcomes. Among the outcomes (Level 4), market reach, customer satisfaction, brand reputation, and employee engagement are modeled as relatively direct effects of capability development, whereas competitive advantage and financial performance are modeled as more distal outcomes that depend on the durability and inimitability of the underlying capabilities.

To make this logic explicit and to support future empirical testing, the framework is summarized in a set of propositions.

***Proposition 1.*** *Comprehensive accessibility across physical, service, communication, and attitudinal domains is positively associated with perceived service quality among guests with accessibility needs.*
***Proposition 2.*** *The relationship between accessibility provision and perceived service quality is mediated by the manner of service delivery, in particular dignity preservation and proactive anticipation.*
***Proposition 3.*** *Inclusive tourism capabilities that are embedded in organizational culture, knowledge systems, and stakeholder relationships are more likely to meet VRIN conditions, and therefore to yield sustained competitive advantage, than capabilities confined to physical infrastructure.*
***Proposition 4.*** *A strategic, rather than compliance-oriented, positioning of inclusive tourism is positively associated with the breadth and durability of competitive advantage.*
***Proposition 5.*** *Dynamic capabilities for continuous improvement strengthen the persistence of inclusive-tourism-based competitive advantage as accessibility standards, technologies, and guest expectations evolve.*
***Proposition 6.*** *The contribution of inclusive tourism capabilities to competitive advantage and financial performance is moderated by destination context, including the level of tourism development, the regulatory environment, and the availability of implementation funding.*

These propositions are not tested in the present study. They are offered as a structured agenda that converts the conceptual framework into statements that subsequent empirical research can examine (see Section 11).

## 4 Pillar one: accessibility management

Accessibility management is the first pillar of an inclusive tourism strategy. It comprises systematic approaches to identifying, removing, and preventing barriers to full participation. This section examines accessibility management across four dimensions: physical, service, communication, and attitudinal accessibility.

### 4.1 Physical accessibility

Physical accessibility concerns the way in which the built environment shapes guests' ability to move through and engage with hotel spaces (40, 41). Comprehensive physical accessibility extends beyond compliance with construction codes to embody universal design principles that make encounters seamless and dignified. Key considerations include accessible entrances with level approaches or gently graded ramps, doors that are easy to operate, adequate clear widths, and appropriate door hardware; interior circulation with corridors wide enough for wheelchair passage, level transitions, slip-resistant flooring, adequate lighting, and visual contrast for guests with low vision; and accessible guest rooms designed around functional needs, including sufficient maneuvering space, appropriate bed height for transfers, accessible bathrooms with roll-in or transfer showers and grab bars, reachable controls, and visual alarm systems.

**Figure 2:**
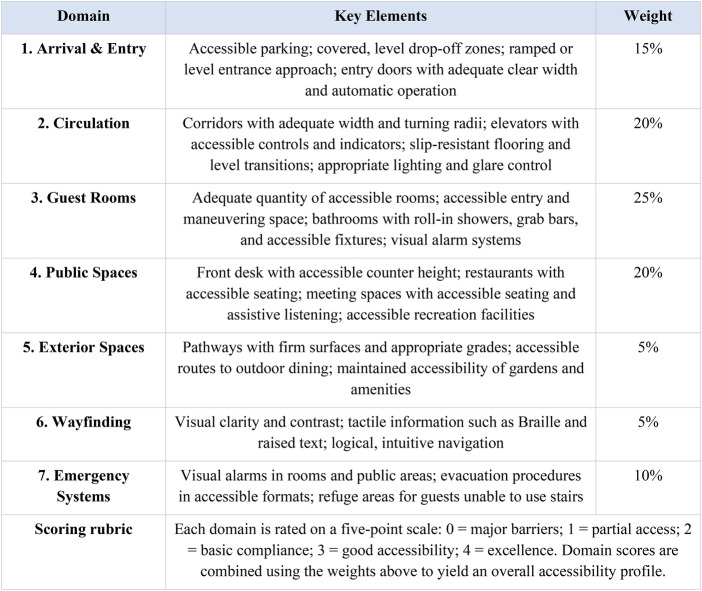
Comprehensive accessibility audit framework: seven-domain assessment with weighted scoring. Authors’ own elaboration based on accessibility standards and universal design principles. The audit framework is proposed as a conceptual planning tool; its weights are illustrative and intended for adaptation to destination priorities, and the instrument requires empirical validation before use as a measurement scale.

Two points warrant emphasis. First, main entrances rather than secondary or service doors should be accessible, since routing guests with mobility needs to separate entrances reinforces stigma. Second, detailed technical parameters, such as specific slopes, widths, and lighting levels, are summarized in the accessibility audit framework (Figure 2) rather than enumerated here; the discussion in this section emphasizes the underlying principle that physical design should enable independent and equitable use rather than merely satisfy minimum dimensions.

### 4.2 Service accessibility

Service accessibility comprises the policies, procedures, and practices that enable all guests to enjoy hotel services and experiences fully (39, 42). Whereas physical accessibility addresses the built environment, service accessibility ensures that an organization's activities can meet a wide range of access needs. Effective service accessibility allows guests to identify accessible room types through detailed descriptions, to request specific accessibility features or assistive equipment, to communicate their access needs and preferences in advance, to receive confirmation that requested accommodations will be available, and to modify accessibility-related reservations without penalty.

Booking systems should follow web design that is easy to use and consistent with the Web Content Accessibility Guidelines, version 2.1 (43). Service accessibility also extends to flexible policies during the stay, since the diversity of access needs cannot always be anticipated through standardized procedures alone.

### 4.3 Communication accessibility

Communication accessibility ensures that guests and hotels can exchange information effectively, regardless of sensory, cognitive, or language differences. For guests who are deaf or hard of hearing, hotels can provide visual alarm systems for fire alarms, doorbells, and telephone calls; text-based communication options; video remote interpreting that connects to sign language interpreters; written versions of spoken announcements; and captioning on televisions and hotel information channels. For guests with vision impairment, hotels can provide information in audio formats, including recorded room tours and spoken descriptions, talking elevators that announce floors, staff trained to give clear verbal directions, and tactile communication such as Braille signage and room numbers, tactile maps, raised-character signs, and textured directional cues.

For guests with cognitive or intellectual disabilities, or guests with limited proficiency in the local language, hotels can use clear, jargon-free language, visual aids such as pictograms and symbols, consistent formats, step-by-step instructions, and confirmation and repetition when requested. Research on the embodied experiences of guests with vision impairment underscores that communication accessibility extends well beyond information provision to include the way in which staff describe, narrate, and frame the surrounding environment (44, 45).

### 4.4 Attitudinal accessibility

Attitudinal accessibility concerns the beliefs, assumptions, and values that shape how staff treat guests with access needs (46–48). Qualitative studies of guests with disabilities consistently report that attitudinal barriers can be at least as consequential as physical ones and often shape the overall evaluation of a stay (49). It is among the most demanding dimensions to address. Negative attitudes appear when staff make assumptions about guests' abilities without asking, adopt a patronizing manner, offer excessive help that undermines independence, hesitate to make reasonable accommodations, display discomfort in interactions, or act on unconscious bias that affects service quality.

Awareness training that addresses the social model of disability, environmental barriers, the diversity of access needs and preferences, the importance of respectful and person-first communication, and the recognition of unconscious bias can support positive attitudes. Interaction training helps staff ask guests directly about their preferences rather than making assumptions, offer assistance in ways that respect autonomy, address guests directly rather than their companions, solve problems collaboratively, and treat all guests with equal respect.

## 5 Pillar two: service quality enhancement

The second pillar of the inclusive tourism strategy addresses service quality, since inclusive tourism excellence requires not only accessible infrastructure but also service delivery that meets the needs of all guests. This section examines service quality through an adapted SERVQUAL model, the management of critical incidents, and systems for service recovery. To address inclusive tourism settings, the traditional SERVQUAL dimensions are adapted and extended. The revised framework retains the five original dimensions and adds two dimensions that are especially relevant to inclusive tourism environments. Table 3 sets out the adapted framework, indicating how each dimension is operationalized for inclusive tourism and the methods through which it may be assessed.

**Table 3:** Adapted SERVQUAL framework for inclusive tourism.

Dimension	Inclusive tourism adaptation	Key indicators	Assessment methods
Tangibles	Physical accessibility features, universal design, assistive equipment	Feature presence, functionality, equipment availability	Physical audits, guest surveys, certifications
Reliability	Consistent accessibility provision, reliable equipment, accurate information	Reliability rates, information accuracy, consistency	Guest feedback, service monitoring, mystery shopping
Responsiveness	Flexible accommodation, proactive problem-solving, timely response	Response times, flexibility, resolution speed	Guest surveys, time tracking, mystery shopping
Dignity Preservation (new)	Maintenance of guest autonomy; avoidance of patronization; respect for independence	Autonomy respect, equal-treatment perception, dignity ratings	Guest surveys, focus groups, observational studies
Proactive Anticipation (new)	Anticipation of needs before requests; preventive problem-solving	Anticipatory service frequency, barrier prevention	Guest feedback, staff initiative tracking, comparative analysis

Authors’ own elaboration adapted from the SERVQUAL framework of Parasuraman et al. (15,29). The dimensions of assurance and empathy from the original model are subsumed within the adapted dimensions above.

The two added dimensions are particularly significant for inclusive tourism. Dignity preservation refers to the importance of respecting guests' independence and avoiding a patronizing manner while still providing needed assistance; it recognizes that the way in which accessibility is facilitated matters as much as the presence of accessibility features. Proactive anticipation refers to recognizing and resolving potential difficulties before guests encounter them, and it represents the highest level of service quality in inclusive tourism settings. As Proposition 2 indicates, these two dimensions are expected to mediate the relationship between accessibility provision and perceived service quality.

Critical incidents are especially important in inclusive tourism settings, where barriers or exceptional accommodations can prompt strong emotional reactions. Empirical work on the lived experiences of visitors with disabilities indicates that a small number of decisive moments — both positive and negative — can disproportionately shape evaluations of the entire stay (45, 50). Positive critical incidents include well-timed assistance, accessibility features that exceed expectations, particularly respectful and dignified interactions, the prompt resolution of problems, and staff recognition of preferences from previous stays. Negative critical incidents include promised accessibility features that are unavailable on arrival, incorrect assumptions or excessive assistance, barriers that prevent the use of advertised facilities, patronizing interactions, and an inability to resolve problems as they arise.

Service failures will occur despite careful planning. In inclusive tourism, where barriers can affect guest well-being, effective service recovery is especially important. Effective recovery involves a prompt response, immediate acknowledgment of the problem, staff who are empowered to respond without waiting for management approval, a sincere apology that accepts responsibility, fair compensation proportional to the impact of the failure, organizational learning, and appropriate follow-up. Service failures affecting accessibility are particularly serious because they can prevent guests from meeting basic needs, compromise dignity, breach accessibility commitments, and create legal exposure under disability rights law.

## 6 Pillar three: competitive advantage through inclusive tourism

**Figure 3:**
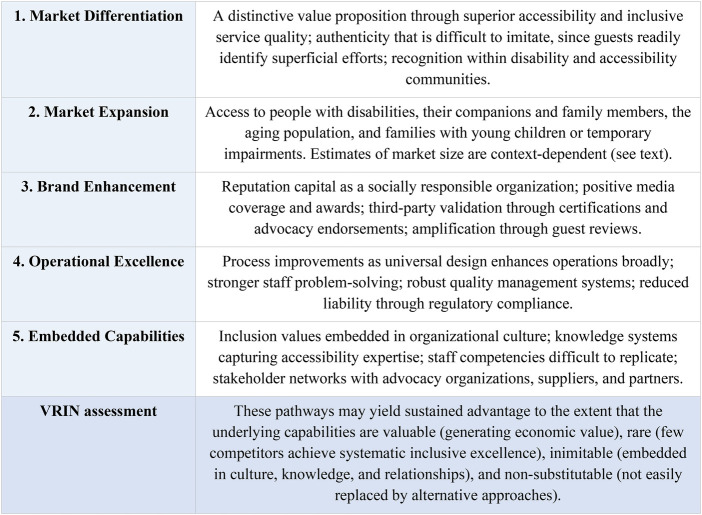
Competitive advantage generation mechanisms: five VRIN pathways. Authors’ own elaboration based on the resource-based view (16) and competitive strategy literature.

The third pillar examines how inclusive tourism practices may generate durable competitive advantage and support strong market positions. Operational excellence often follows from implementing inclusive practices, and these practices can lead to broader operational gains. Universal design benefits all guests, not only those with specific access needs; staff training can improve service quality and problem-solving capacity; process improvements can enhance efficiency and reliability; and quality management systems can strengthen overall performance. Studies of hotels that have invested in accessibility report cross-cutting benefits, including process improvements that also serve guests without specific access needs (41, 51). Recent work on mega-event legacies suggests that investments in more sustainable and accessible hotel operations may yield lasting operational improvements beyond the original implementation phase (52). Inclusive tourism can also act as a catalyst for innovation, since addressing diverse needs encourages solutions that are transferable to other settings and can attract partnerships with assistive technology providers. Figure 3 summarizes five VRIN-aligned pathways through which inclusive tourism capabilities may translate into competitive advantage.

The market expansion potential of inclusive tourism is best understood through market segmentation and value-based pricing theory (53, 54). Inclusive provision addresses identifiable and partially underserved segments, including travelers with disabilities, their companions, senior travelers, and families with young children. Where some guests are willing to pay a premium for accessible offerings, this reflects the value they place on reliable accessibility and dignified service rather than an arbitrary surcharge. The magnitude of this potential is not uniform: it is moderated by destination income levels, the regulatory environment, the degree of existing competition for accessible segments, and prevailing cultural attitudes toward disability (6). Estimates of market size and premium pricing should therefore be treated as context-dependent rather than universal.

Hotels can adopt different positioning strategies for inclusive tourism. An accessibility-leader positioning treats inclusive tourism as a primary strategic differentiator, supported by substantial investment across physical, service, communication, and attitudinal accessibility, active marketing to accessible segments, partnerships with advocacy organizations, continuous innovation, and strong measurement systems. A compliance-oriented positioning focuses mainly on meeting minimum requirements and reducing risk; it lowers investment but also limits competitive advantage and may prove insufficient as regulation tightens and guest expectations rise. As Proposition 4 indicates, a strategic rather than compliance-oriented positioning is expected to be associated with broader and more durable advantage.

Sustained advantage requires continuous improvement, since static excellence is insufficient when standards change, technologies develop, and competitors improve. Advantage is more durable when capabilities are embedded across skills, culture, knowledge management systems, staff attitudes, routines, physical infrastructure, and partner relationships. Consistency over time, alignment between marketing promises and actual delivery, third-party validation, positive guest reviews, and industry recognition together build authenticity and trust. For strategic coherence, an inclusive tourism strategy must align with the organization's overall strategy, brand positioning, service quality objectives, values, and resource allocation. Table 4 sets out an illustrative performance measurement framework that links these strategic considerations to market, customer, brand, and financial indicators.

**Table 4:** Performance measurement framework.

Dimension	Key metrics	Data sources	Illustrative target levels
Market Performance	Market share, booking growth, geographic penetration	Reservation systems, market research	Growth in accessible-segment bookings; rising market share
Customer Satisfaction	Overall satisfaction, NPS, accessibility satisfaction, return rate	Guest surveys, online reviews, CRM data	High satisfaction and NPS; strong return rate
Brand Reputation	Brand awareness, media coverage, awards, social sentiment	Brand tracking, media and social analytics	Improved awareness and positive sentiment
Financial Performance	Segment revenue, premium pricing, RevPAR, margins	Financial systems, revenue management	Revenue growth; sustainable premium where context permits

Authors’ own elaboration based on balanced scorecard methodology and hospitality performance metrics. Target levels are presented as illustrative planning benchmarks rather than prescriptive thresholds; appropriate targets depend on destination context, property type, and baseline performance.

## 7 Contextual boundaries and conditions of application

The ITSF is presented as a general framework, but its application is conditioned by context. Three sets of contextual factors are particularly important.

First, destination development context matters. The level of tourism development, the maturity of supporting infrastructure such as transport, public space, and attractions, and the strength of the regulatory environment vary widely across destinations. In mature destinations with established accessibility regulation, hotels may build inclusive capabilities on top of an already accessible destination system. In emerging destinations, hotels may need to compensate for gaps in the surrounding environment, which raises the cost and complexity of implementation. The availability of public support, including grants, tax incentives, and low-interest financing, also differs markedly and directly affects which implementation pathways are feasible.

Second, organizational context matters. Large hotel chains can spread investment across many properties, draw on centralized expertise, and embed accessibility in brand standards, whereas small and independent hotels typically face tighter capital constraints and thinner managerial capacity. Property type is also relevant, since urban business hotels, resorts, and heritage properties differ in their physical adaptability and in the accessibility expectations of their guests.

Third, socio-cultural context matters. Prevailing attitudes toward disability, the strength of disability advocacy, and the degree to which accessibility standards reflect local rather than imported assumptions all shape both the demand for inclusive tourism and the meaning of dignified service in a given setting. These socio-cultural factors interact with broader sustainability and responsibility agendas in tourism and should be read alongside calls for context-sensitive responsibility frameworks (55).

The ITSF is most directly applicable to hotels operating in destinations with at least a developing accessibility infrastructure and a supportive or strengthening regulatory environment. In other contexts, the framework remains useful as a structuring device, but its capability domains, implementation sequencing, and performance benchmarks should be adapted to local conditions. Proposition 6 formalizes destination context as a moderator of the relationship between inclusive capabilities and competitive outcomes, and comparative research across destination types is needed to specify these boundary conditions more precisely. Embedding the framework within a specific destination, with attention to its development stage and funding landscape, is likely to surface practical, context-specific considerations that a general model cannot anticipate.

## 8 Implementation considerations

**Figure 4:**
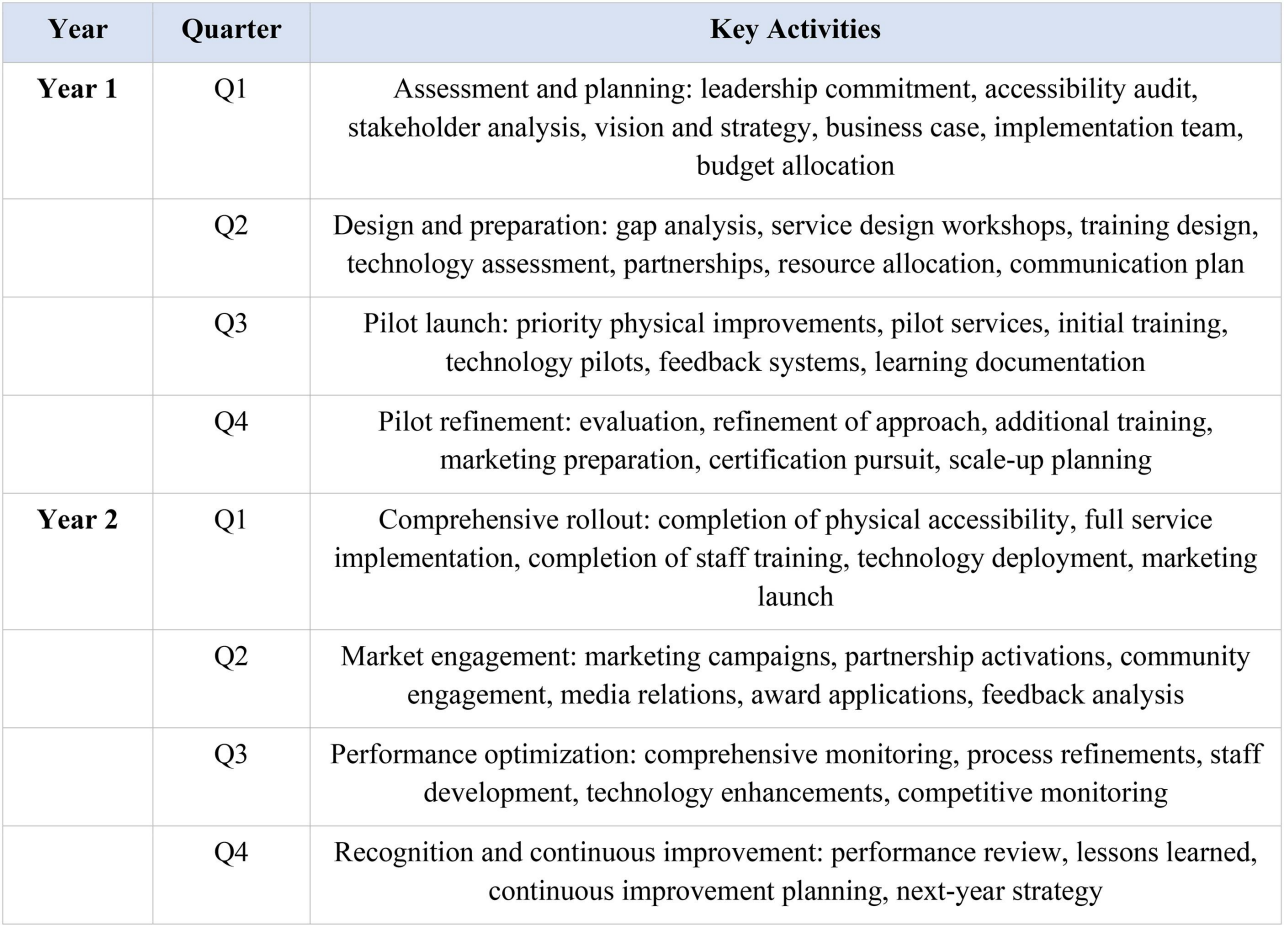
Implementation roadmap. Authors’ own elaboration based on change management and hospitality implementation literature.

Implementing inclusive tourism requires organizational change and therefore a methodical approach to change management (56). A staged approach can be organized into five phases. Phase 1, building awareness (months 1 to 3), educates leaders, analyzes stakeholders, assesses the current state of accessibility, develops a vision, prepares a business case, and plans stakeholder communication. Phase 2, planning and design (months 4 to 6), conducts detailed accessibility audits, holds service design workshops, assesses technology needs, designs training, sequences implementation, allocates resources, and develops partnerships. Phase 3, pilot implementation (months 7 to 12), tests new methods in small areas, trains initial staff groups, makes priority physical improvements, redesigns service processes, deploys initial technology, and collects feedback. Phase 4, scaled implementation (months 13 to 24), rolls out successful pilots, completes staff training and physical accessibility, integrates accessibility across organizational systems, launches marketing, and monitors performance. Phase 5, continuous improvement (month 25 onward), maintains monitoring, integrates guest feedback, adopts new technologies, develops staff, benchmarks against best practice, and pursues recertification. Figure 4 maps these phases onto a quarter-by-quarter implementation roadmap.

Organizations face recurring challenges when pursuing inclusive tourism. Resource constraints arise because improvements in physical accessibility require capital that competes with other priorities; these can be mitigated through staggered implementation, integration with renovation cycles, the use of grants and tax incentives, and prioritization of the most cost-effective changes. Knowledge gaps arise from limited familiarity with universal design and implementation practice; these can be mitigated through external expertise, staff training, benchmarking, and participation in industry associations. Research on marketing communication that respects sustainability criteria in the tourism sector offers useful foundations for addressing such knowledge gaps and for raising the visibility of inclusive tourism initiatives (57, 58).

## 9 Strategic implications of the ITSF

The ITSF carries implications for actors beyond the individual hotel. Because the framework specifies capability domains and an accessibility audit logic, it can inform policy and education in concrete ways.

For regulators and tourism authorities, the ITSF suggests that regulation setting minimum accessibility standards is necessary but not sufficient. The framework's distinction between compliance-oriented and strategic positioning implies that regulation should be paired with positive incentives that reward provision beyond the minimum. The capability domains indicate where such incentives are most needed, namely not only physical infrastructure but also staff competency, technology, and quality assurance. The accessibility audit framework in Figure 2, although developed as a managerial tool, also offers regulators a structured and weighted basis for standard-setting and monitoring, and its domain weights can be adjusted to reflect destination priorities. Incentive instruments such as tax credits, grants for small independent hotels, low-interest financing, and recognition programs map directly onto the resource allocation mechanism in Level 3 of the framework and address the funding constraints identified in Section 7.

For tourism educators, the ITSF's capability domains provide a template for curriculum design. Rather than treating inclusive tourism as a stand-alone elective, programs can map accessibility content onto existing courses in operations, marketing, finance, strategy, and human resources, mirroring the cross-functional capability structure of the framework. The audit logic and the extended SERVQUAL model offer concrete teaching tools for developing students’ ability to assess accessibility and inclusive service delivery.

For public awareness efforts, the framework's emphasis on attitudinal accessibility and on stakeholder engagement implies that awareness campaigns are not peripheral but form part of the capability system itself. Campaigns that raise the visibility of accessible offerings, disseminate implementation models, and strengthen demand contribute directly to the market expansion outcomes the framework identifies.

**Figure 5:**
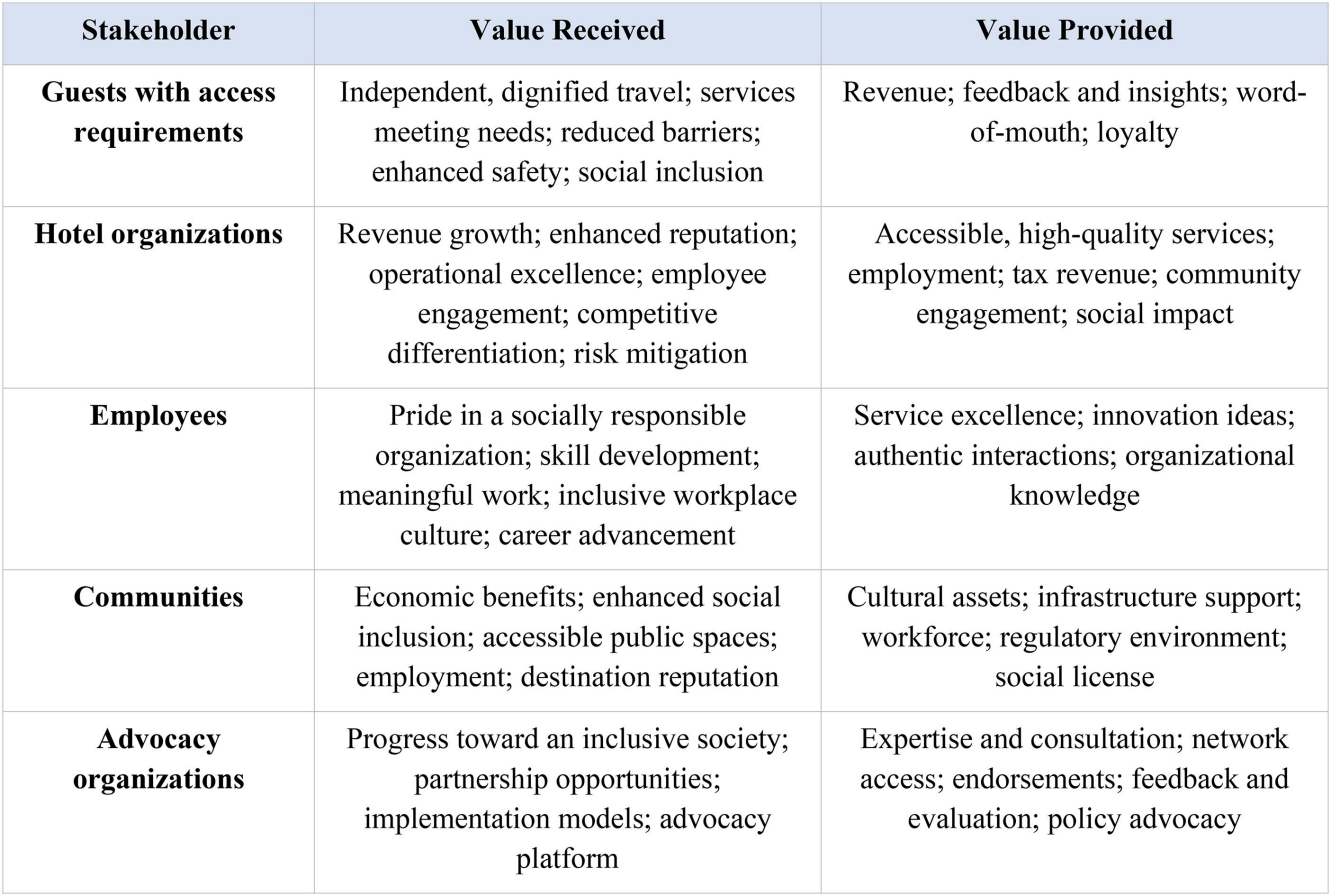
Stakeholder value creation map. Authors’ own elaboration based on stakeholder theory (35) and inclusive tourism literature.

The framework also reinforces the value of comprehensive performance measurement. Because the ITSF links capabilities to multiple outcome categories, measurement spanning financial, operational, customer, employee, and stakeholder dimensions allows evidence-based decisions, supports accountability, and enables the feedback loops on which continuous improvement depends. Figure 5 maps the value flows that the framework anticipates among the principal stakeholders.

## 10 Discussion

### 10.1 Theoretical and practical contributions

This conceptual study contributes to hospitality management and tourism studies in several ways. It integrates strategic management, service quality, accessibility studies, and disability research, which are usually examined separately, into a single framework, thereby clarifying connections across disciplines. It extends service quality theory by adapting SERVQUAL to include dignity preservation and proactive anticipation, dimensions that may also be relevant to other service settings involving vulnerable or underserved groups. It articulates the mechanisms through which inclusive tourism may generate competitive advantage, extending the resource-based and dynamic capabilities perspectives to this context and arguing that social responsibility and competitiveness need not be in tension. Finally, the implementation framework addresses the gap between conceptual models and practice, since many accessible tourism frameworks specify what is required without specifying how it can be achieved.

For practitioners, the framework offers a roadmap with step-by-step guidance on assessing accessibility, sequencing implementation, building capabilities, and monitoring performance. The business-case elements help managers articulate value creation and competitive benefits, and the figures and tables function as practical planning tools.

### 10.2 Limitations

As a conceptual study, this paper has several limitations. First, the ITSF and its propositions have not been empirically validated; the relationships it specifies are theoretically argued expectations rather than demonstrated findings. Second, several of the accessibility standards on which the framework draws originate largely in high-income, Western regulatory contexts and may embed assumptions that do not transfer directly to all destinations. Third, the framework is built from a deliberately selected set of theoretical lenses; other lenses, such as institutional theory or service-dominant logic, could yield additional or alternative insights. Fourth, as discussed in Section 7, the framework's generalizability is bounded by destination, organizational, and socio-cultural context. Finally, the quantitative benchmarks included in the performance framework are illustrative planning aids and are not derived from empirical norms. These limitations define the conditions under which the framework should be interpreted and applied, and they motivate the research agenda set out below.

## 11 Future research

This study provides a conceptual foundation for several lines of future research. Rather than restating a general need to understand inclusive tourism, the agenda below identifies research questions that follow directly from the framework and propositions developed here.

The propositions in Section 3 offer a starting point for empirical validation. Propositions 1 and 2 invite quantitative studies, including survey-based and structural models, that test the links among accessibility provision, the manner of service delivery, and perceived service quality. Propositions 3 to 5 invite longitudinal and qualitative work examining whether inclusive capabilities embedded in culture and relationships are in fact more durable, and how dynamic capabilities sustain advantage as standards evolve. Proposition 6 calls specifically for comparative, multi-country research that treats destination context as a moderator and that specifies the boundary conditions of the framework.

Implementation research can use the ITSF's four-level architecture as an analytical scaffold, examining which capability domains are most decisive, which implementation mechanisms are most effective, and how the roadmap performs in practice. The audit framework and the extended SERVQUAL model provide candidate instruments whose reliability and validity should themselves be tested and refined. Guest experience research can build on the four-dimensional model of accessibility to investigate the relative importance of the physical, service, communication, and attitudinal dimensions, and the role of critical incidents and service recovery, across and within different groups of guests with access needs.

Methodologically, this agenda would benefit from mixed-methods designs, multi-country comparative case studies, and longitudinal evaluations of ITSF implementation, which together would allow both the testing of propositions and the contextual refinement of the framework.

## 12 Conclusion

This conceptual study examined strategic hotel management for inclusive tourism and developed the Inclusive Tourism Strategic Framework (ITSF), which integrates accessibility management, service quality enhancement, and the generation of competitive advantage. Its central argument is that inclusive tourism is a strategic opportunity, not solely a matter of legal compliance or social responsibility, and that hotels developing strong inclusive capabilities are likely to achieve wider market reach, higher guest satisfaction, stronger brand reputation, greater employee engagement, and more durable competitive advantage.

The framework's main contribution is to connect three areas that are usually examined separately. It treats comprehensive accessibility across physical, service, communication, and attitudinal dimensions as a foundation; it extends service quality theory with the dignity preservation and proactive anticipation dimensions; and it specifies, through capability domains and implementation mechanisms, how hotels can build and sustain inclusive capabilities that are difficult for competitors to replicate. Through a set of propositions and a discussion of contextual boundaries, the framework also makes explicit both the relationships it expects and the conditions under which it is most applicable.

As populations age and expectations of accessible service rise, hotels face a strategic choice between proactively embracing inclusive tourism, pursuing minimal compliance, or resisting change. The ITSF and the accompanying research agenda are intended to support the first of these paths and to provide a foundation for the empirical work needed to test, refine, and contextualize the framework.
